# Attosecond chronoscopy of the photoemission near a bandgap of a single-element layered dielectric

**DOI:** 10.1126/sciadv.ado0073

**Published:** 2024-06-26

**Authors:** Dionysios Potamianos, Maximilian Schnitzenbaumer, Christoph Lemell, Pascal Scigalla, Florian Libisch, Eckhard Schock-Schmidtke, Michael Haimerl, Christian Schröder, Martin Schäffer, Johannes T. Küchle, Johann Riemensberger, Karl Eberle, Yang Cui, Ulf Kleineberg, Joachim Burgdörfer, Johannes V. Barth, Peter Feulner, Francesco Allegretti, Reinhard Kienberger

**Affiliations:** ^1^ Physik Department, Technische Universität München, Garching, 85748, Germany.; ^2^ Institute for Theoretical Physics, Vienna University of Technology, Vienna, 1040, Austria.; ^3^ Laboratory of Photonics and Quantum Measurements, École Polytechnique Fédérale de Lausanne, Lausanne, CH-1015, Switzerland.; ^4^ Max-Planck Institut für Quantenoptik, Garching, 85748, Germany.; ^5^ Fakultät für Physik, Ludwig-Maximilians-Universität München, Garching, 85748, Germany.

## Abstract

We report on the energy dependence of the photoemission time delay from the single-element layered dielectric HOPG (highly oriented pyrolytic graphite). This system offers the unique opportunity to directly observe the Eisenbud-Wigner-Smith (EWS) time delays related to the bulk electronic band structure without being strongly perturbed by ubiquitous effects of transport, screening, and multiple scattering. We find the experimental streaking time shifts to be sensitive to the modulation of the density of states in the high-energy region (*E* ≈ 100 eV) of the band structure. The present attosecond chronoscopy experiments reveal an energy-dependent increase of the photoemission time delay when the final state energy of the excited electrons lies in the vicinity of the bandgap providing information difficult to access by conventional spectroscopy. Accompanying simulations further corroborate our interpretation.

## Teaser

Photoionization timing is exploited for detecting otherwise inaccessible high-energy region band structure features.

## INTRODUCTION

Photoelectron spectroscopy (PES), particularly with angle and spin resolution (ARPES and SARPES), is the standard technique for studies of the electronic properties of even highly complex materials (*1*), including spin textures of topological and Rashba-type (*2*, *3*), as well as ferroelectric, samples (*4*). With the advent of laser techniques reaching the attosecond domain, the temporal resolution of photoemission, one of the fastest processes in matter, has attracted considerable interest (*5*). Pump-probe techniques with attosecond precision phase-synchronized laser pulses are the methods of choice for such studies (*5*–*10*), although other techniques are applicable as well, e.g., combining synchronized soft x-ray pump and circular-polarized infrared (IR) probe pulses [angular streaking (*11*)] in single shot experiments (*12*). Even photoemission without time resolution can supply valuable complementary insight, e.g., the Eisenbud-Wigner-Smith (EWS) delays obtained for the sp-band emission from Cu(111) by time-independent SARPES (*13*), the delays of the highest occupied molecular orbital (HOMO) and HOMO-1 photoemission from C_60_ at the 20-eV surface plasmon resonance extracted from ARPES data (*14*), and the transport delays by resonant versus nonresonant photoemission in solids estimated from resonance widths (*15*, *16*).

Attosecond pump-probe experiments have succeeded in directly measuring time delays between photoelectrons emitted from different initial states. For the gas phase, reduction of experimental uncertainties down to the single attosecond range was achieved (*17*). For light atoms like He or Ne agreement between experiment and theory is excellent, both for main lines and shake-up satellites (*17*–*19*). In particular, helium, for which the photoemission timing can be calculated exactly (*17*), proved to be an ideal calibration standard for the measurement of absolute emission delays, i.e., the time elapsing between photon absorption and electron emission. Heavier atoms for which no full ab initio calculations are possible have also been used as emission time standards (*20*). Using atoms in the gas phase as chronoscopic reference, the first measurement of the absolute time delay between photon absorption and electron emission in condensed matter has been achieved (*20*, *21*).

Since the first realization of attosecond photoelectron emission from a solid surface (*22*), many aspects of ultrafast photoemission dynamics have been addressed by experiments and theory: effects of electron localization (*23*, *24*) and excitation of intrinsic plasmons (*25*), delays of resonant versus nonresonant photoemission (*16*, *26*, *27*) including contributions from surface states (*21*, *27*), the role of the effective mass (*16*, *28*), the influence of scattering, screening (*29*, *30*), and of the orbital angular momentum of the initial (*31*) and final states (*32*), and transport times through adlayers of well-defined thickness (*21*, *33*).

In attosecond PES, usually only time differences between groups of electrons, e.g., from different initial states, are measured. Comparison between numerically exact simulations and experimental data has enabled insight into the absolute timing of processes involved in the emission of photoelectrons (*5*, *20*, *21*), which strongly depends on the potential landscape at the point of origin as shown for photoemission from within a C_60_ cage (*5*, *34*).

Extraction of detailed dynamical information from time-resolved photoemission from solid surfaces is challenging because of the difficulty to disentangle the complex interplay between atomic properties of electrons such as their angular momentum (*5*, *31*) and the unperturbed band structure from electron transport and scattering (*35*). As the timing information of the photoemission is extracted from either time shifts in streaking traces (*7*, *11*, *12*) or phase shifts in reconstruction of attosecond beating by interference of two-photon transitions (RABBITT) interferences (*6*) induced by the probing IR field, attosecond PES from metals effectively probes the arrival time of the emitted electron at the surface and in vacuum (*5*, *22*, *36*) because of the efficient sub–angstrom-scale screening of the IR field in the bulk (*5*, *31*). The arrival time therefore represents a convolution of many competing processes and can provide only indirect information on dynamical processes and the band structure in the bulk (*24*, *36*, *37*).

The present study aims at overcoming some of these limitations by exploring the chronoscopy of a layered material for which the anisotropic dielectric screening of the IR probing field in the direction perpendicular to the layers is strongly suppressed. Hence, the influence of the bulk band structure on the timing of the photoelectron becomes directly accessible.

## RESULTS

One fundamental observable of interest is the EWS scattering time delay $\tau_{\text{EWS}}=-\frac{i}{2}\left\langle S^{+}\;|\;\frac{\partial }{\partial E}\;|\;S\right\rangle$ (**S**, scattering matrix; *E*, energy) which for the half-scattering scenario of photoemission characterizes the time it takes to form the outgoing wave packet upon absorption of the ionizing extreme ultraviolet (XUV) photon. τ_EWS_ sensitively probes the final state in the band structure accessed by photoabsorption (*5*). In the present case of XUV photons, the scattering final state lies in the high-energy region of the solid-state band structure ≳80 eV above the Fermi edge. Investigations of the EWS time delays therefore offer an avenue to access properties of this region of the band structure. Of particular interest are strong modulations of the (projected) band structure near Brillouin zone boundaries, which are difficult to extract from spectroscopy in this energy domain because of the convolution with the occupied ground-state band structure. First measurements suggested delayed arrival times at the surface due to Bragg reflection (*22*). Theoretical investigations using inverse low-energy electron diffraction (LEED) states (*37*, *38*) suggested a substantial reduction of time delays near bandgaps. However, an unambiguous assignment was difficult because of the presence of a host of competing processes.

**Fig. 1.**
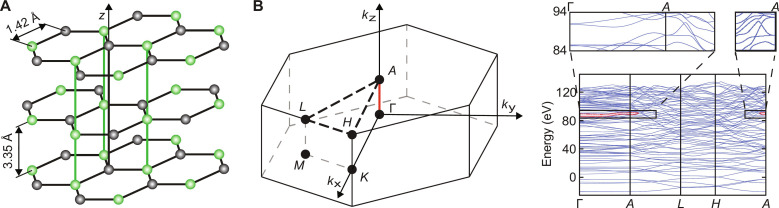
Properties of HOPG. (**A**) Crystal structure of HOPG: Honeycomb structure in the *xy* plane and layer stacking in the *z* direction (along the crystallographic $\hat{c}$ axis) with a direct neighbor of every second atom (*39*, *40*, *42*). (**B**) Brillouin zone and band structure of HOPG calculated using the Vienna Ab initio Simulation Package (VASP) (this calculation in local density approximation, cutoff energy *E*_cut_ = 120 eV, 30 × 30 × 8 *k*-points and a large number of iterations to assure convergence of high-energy states). The kinetic energy axis has been scaled according to the electron reflectivity measurements (see Materials and Methods). The bandgap within the investigated final state energy range is highlighted in red and focused at the insets. It covers the Γ − *A* direction, i.e., the direction parallel to the normal of the (0001) surface including a cone of ±15° around it.

We use in this work the archetype of single-element layered samples, highly oriented pyrolytic graphite (HOPG). It consists of stacked graphene monolayers, laterally offset from each other in an ABAB pattern, such that only every second carbon atom has a direct neighbor in *z* direction (see Fig. 1A) (*39*, *40*). The rigidity of the graphene monolayers guarantees a well-defined layer spacing of $d=3.35\overset{\cdot }{A}$ over the whole target. Small variations of the layer spacing at the surface (*41*) only slightly affect the position of the bandgaps, resulting in a potential landscape in the double-layer unit cell with a well-defined periodicity. Accordingly, Bragg scattering along the direction of the surface normal is expected at wave numbers corresponding to integer multiples *n* of the distance from the center (Γ) to the boundary (A) of the Brillouin zone ΓA=π2d with bandgaps at energies *E_n_* = *n*^2^ 0.838 eV. Typically, bandgap separations and associated modulations of ρ(*E*) become smaller for larger *n*. For HOPG, however, density functional theory (DFT) calculations optimized for convergence at large energies indicate a wide bandgap at *E*_gap_ ~ 84 eV above Fermi level (for additional details, see the Supplementary Materials). This is very close to the predicted gap near the excitation energy *E*_*n*=11_ relative to the effective background potential in HOPG of 17.17 eV (*42*).

The bandgap (red-shaded area in Fig. 1B) appears not only pronounced along the direction normal to the surface (ΓA) but also extends to about ~15° into the off-normal directions, i.e., it covers also the acceptance angle of our electron detector (±2°) when mounted with its axis perpendicular to the surface and with the electrostatic einzel lens turned off (see below). Accordingly, the influence of the bandgap is expected to be clearly visible in our attosecond chronoscopy experiment performed with photon energies (*E*_XUV_) ranging from 80 to 130 eV. Most important in the present context is the highly anisotropic dielectric response of HOPG (*43*, *44*), which allows penetration of a near IR (NIR) field oriented perpendicular to the atomic layers. The streaking field therefore immediately clocks the wave packet upon its formation in the high-energy band structure and, thus, provides direct access to the corresponding EWS delay. Transport contributions to the emission delay, while still present, are reduced compared to emission from metals.

**Fig. 2.**
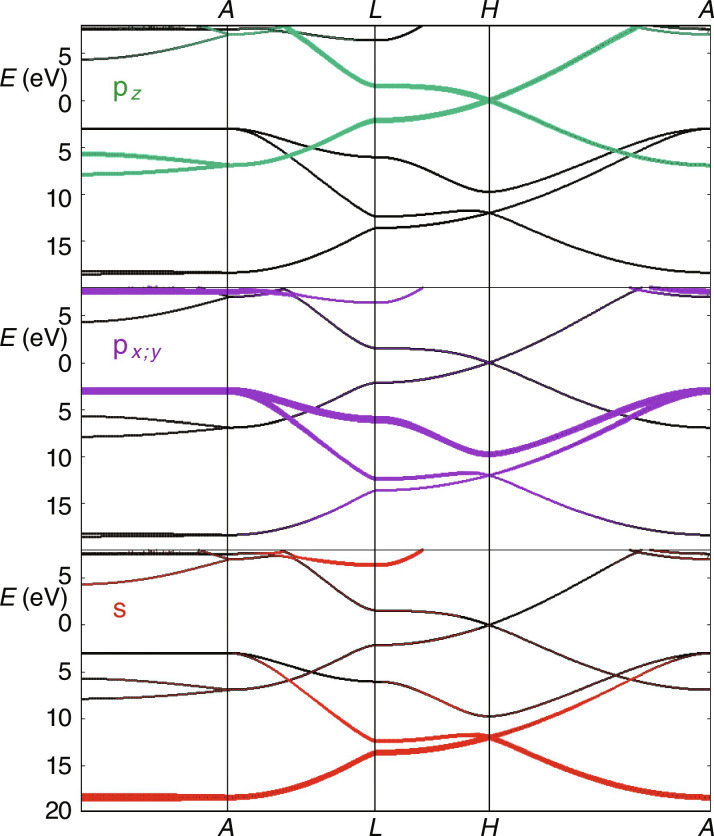
DFT calculations of HOPG band structure in the vicinity of the ground state. C2s, C2p*_xy_*, and C2p*_z_* are the contributions to the valence band of HOPG. The lower HOPG valence bands have mainly C2s character (red), while the upper bands have mainly contributions from C2p (purple and green). The Fermi energy of HOPG is set at *E* = 0 eV.

Previous experimental streaking studies exploited emission from atomic core levels as reference for the relative timing of valence band photoemission. HOPG does not feature core level photoemission for photon energies below the C1s absorption edge at 284 eV. However, DFT band structure calculations of the HOPG valence band [Fig. 2; see also (*45*)], x-ray photoelectron spectroscopy (XPS), as well as x-ray emission spectroscopy studies (*46*) show a structured valence band spectrum with the more strongly bound part mainly derived from atomic 2s states and the parts closer to the Fermi level mainly from atomic 2p states. The centers of gravity of the two distributions (⟨*E*_s_⟩ and ⟨*E*_p_⟩) are separated by about 15 eV. This feature enables the discrimination of photoelectrons excited from p-derived states and s-derived states even with our spectrally broad XUV pulses of 3.5 to 5 eV full width at half maximum (FWHM). Thus, measurement of the relative timing between p-derived versus s-derived electrons from within the valence band using the attosecond streaking spectroscopy technique is achieved.

**Fig. 3.**
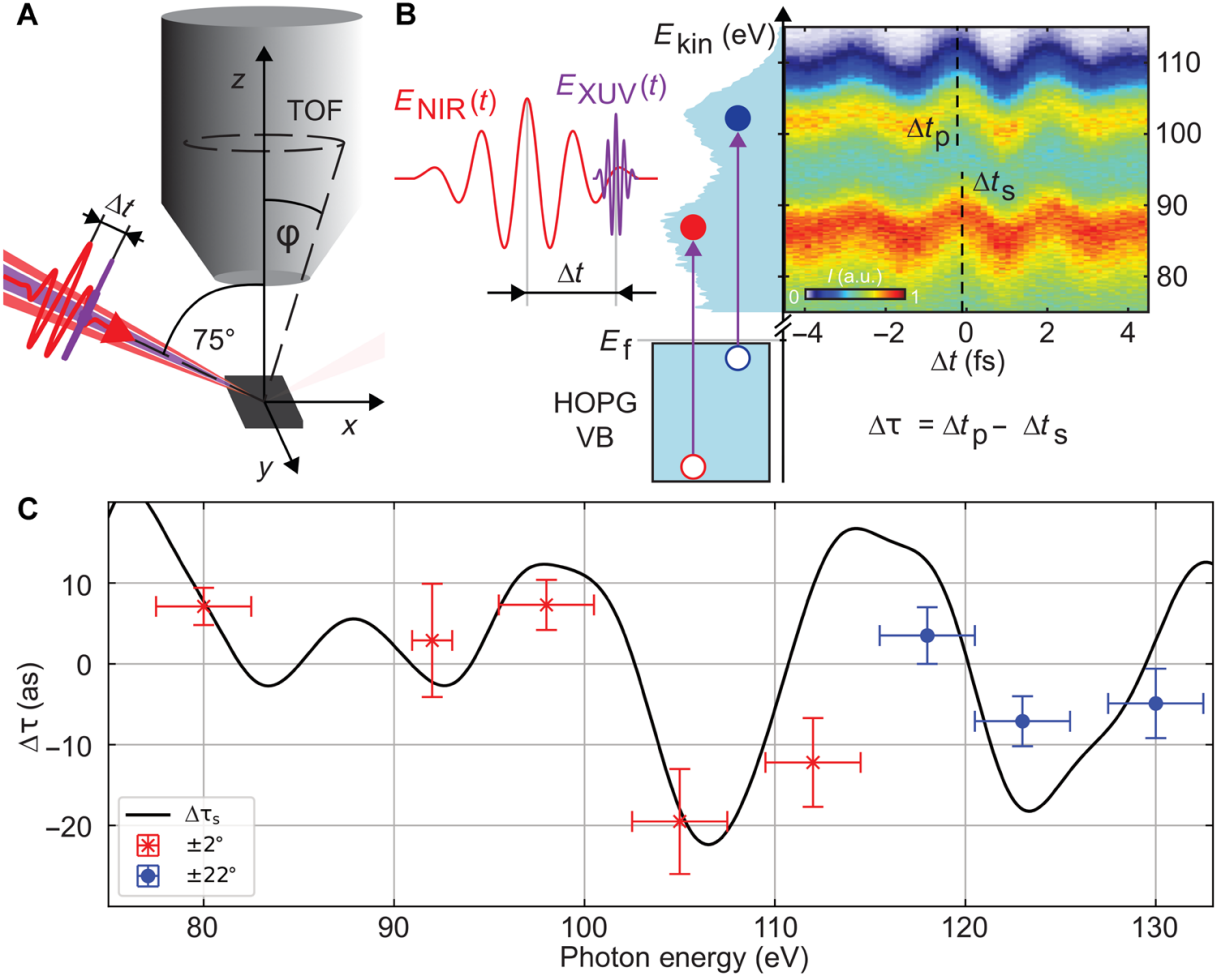
Attosecond chronoscopy of HOPG. (**A**) Geometry of the attosecond photoemission experiments. The attosecond XUV pulse (purple) and the NIR streaking field (red) are focused on the HOPG surface at an angle of incidence of 75°. The photoelectrons emitted perpendicular to the sample surface are detected by a TOF spectrometer. The preset detection acceptance angle ϕ is ±2°, which can be increased to ±22° by using an electrostatic lens when the number of electron counts is diminished due to low flux at higher XUV photon energies. (**B**) Schematic illustration of the attosecond streaking spectroscopy technique. The two laser pulses, XUV pulse (purple) and NIR streaking field (red), are shifted in time with respect to each other (∆*t*). This causes the electrons released by the XUV pulse (red: s-derived peak; blue: p-derived peak) to be clocked by the streaking field. The stationary photoelectron spectrum in the absence of the streaking field (left, light blue) and the streaking traces were measured at a photon energy of 112 eV. (**C**) Experimental data for acceptance angles 2° (red crosses) and 22° (blue dots), respectively, with error bars corresponding to confidence levels of 95% and simulation of the relative streaking time delay ∆τ_s_ between p-electrons and s-electrons of the HOPG valence band as a function of excitation energy (black solid line, see text).

For the present attosecond chronoscopy experiments, the geometric arrangement shown in Fig. 3A was used. Here, the angle of incidence of the two P-polarized beams (NIR pulse and XUV pulse) was 75° with respect to the surface normal. The time-of-flight (TOF) spectrometer was positioned perpendicular to the sample surface. The characteristic streaking traces were measured by detecting the photoelectrons with acceptance angles ϕ of ±2° and ±22° depending on the setting of the TOF’s electrostatic einzel lens. In Fig. 3B, a typical photoemission spectrogram (for *E*_XUV_ = 112 eV) of the HOPG valence band is shown, with the characteristic p- and s-electron peaks at ~103 and ~89 eV, respectively. From such spectrograms, the relative time delay between the two streaking traces was retrieved by the restricted differential time-dependent Schrödinger equation technique (*27*, *47*). This retrieval algorithm was selected because of its superior background rejection properties compared to other available algorithms, such as frequency resolved optical gating (FROG)–based and ptychography-based ones (*17*, *48*–*51*).

As the XUV photon energy is increased, the p-derived electrons from the HOPG valence band with mean energy ⟨*E*_p_⟩ reach the bandgap at *E*_gap_ = 84 eV at a photon energy of *E*_XUV_ ≈ 90 eV, while the s-derived component with mean energy ⟨*E*_s_⟩ reaches the bandgap at *E*_XUV_ ≈ 105 eV. The observed relative photoemission delay ${\Delta \tau }_{s}=\tau_{s}^{\left(p\right)}-\tau_{s}^{\left(s\right)}$ displays pronounced modulations (Fig. 3C). Because of diminishing photoemission cross sections and XUV generation efficiency, photoemission time delays above *E*_XUV_ = 112 eV could only be determined using the larger acceptance angle (ϕ = ±22°) of the TOF. To assess the influence of the acceptance angle on the photoemission delay, a smaller set of measurements at *E*_XUV_ ≈ 98 eV and *E*_XUV_ ≈ 105 eV was also performed with the wide-angle mode, for which deviations from the small-angle mode were below experimental scatter.

The energy-dependent modulation with a pronounced minimum at 105 eV and local maxima near 98 and 118 eV is in good agreement with theoretical predictions for the relative time delay between the p- and the s-contributions to photoemission without invoking any freely adjustable parameters. These simulations are based on the interrelation between the EWS time delay and the modulation of the density of states (DOS) ∆ρ(*E*), τ_EWS_(*E*) = 2π∆ρ(*E*) (see also the Supplementary Materials) (*52*). This connection, related to the Friedel sum rule (*53*–*58*), is also closely related to the Heisenberg time of complex quantum dynamics (*59*, *60*) characterizing the time required for the spread of an initially localized wave packet over a finite irregularly shaped potential landscape. In the present case, it describes the spread of the photoionization wave packet over the real space crystalline unit cell. From a full 3d DFT band structure calculation, we determine the (projected) high-energy DOS from which the difference in the time $∆\tau_{s}=\tau_{s}^{\left(p\right)}-\tau_{s}^{\left(s\right)}\propto \rho \left(E_{\text{XUV}}-\left\langle E_{p}\right\rangle \right)-\rho \left(E_{\text{XUV}}-\left\langle E_{s}\right\rangle \right)$ can be derived. Because of the close energetic proximity of ⟨*E*_p_⟩ and ⟨*E*_s_⟩, $\frac{\text{∆}E}{E}∼10\%$ , at present, XUV energy (~100 eV) corrections due to Coulomb-laser coupling (CLC) and transport are nearly identical for the p- and s-derived electrons and do not significantly contribute to the time difference ∆*t_s_*. The absolute CLC-induced time delay for energetic electrons is τ_CLC_ ≤ 5 as and can be neglected. Estimates of the inelastic mean free path (IMFP) of emitted electrons from the valence band in the energy window of the experiment (*E*_kin_ ~ 100 eV) range from λ_vb_ ~ 8.5 atomic units (au) for amorphous carbon (*61*) to λ_vb_ ~ 16.75 au for the emission along the direction normal to the atomic planes of HOPG (*62*). Residual transport effects contributing to the absolute time delays are included in the simulation (see the Supplementary Materials). Alternatively, to exploiting the interrelation between DOS modulations and τ_EWS_, the EWS delay can be directly calculated from the scattering phases of inverse LEED states. We demonstrate the connection between DOS variations and the spectral variation of scattering phases for a one-dimensional model of HOPG, taking into account the layer structure in $\hat{c}$ direction with a lattice spacing of d=3.354Å . A more detailed description can be found in the Supplementary Materials.

The present findings suggest that time delay measurements allow the unambiguous localization and identification of bandgaps through the associated modulation of the DOS of HOPG even at high energies (see Fig. 3C). Identifying such structures in the high-energy continuum would pose a considerable challenge in the spectral domain as in the absence of a time marker the convolution of the initial and final densities of states in the joint DOS would most likely mask this structure (see also Fig. 3B).

**Fig. 4.**
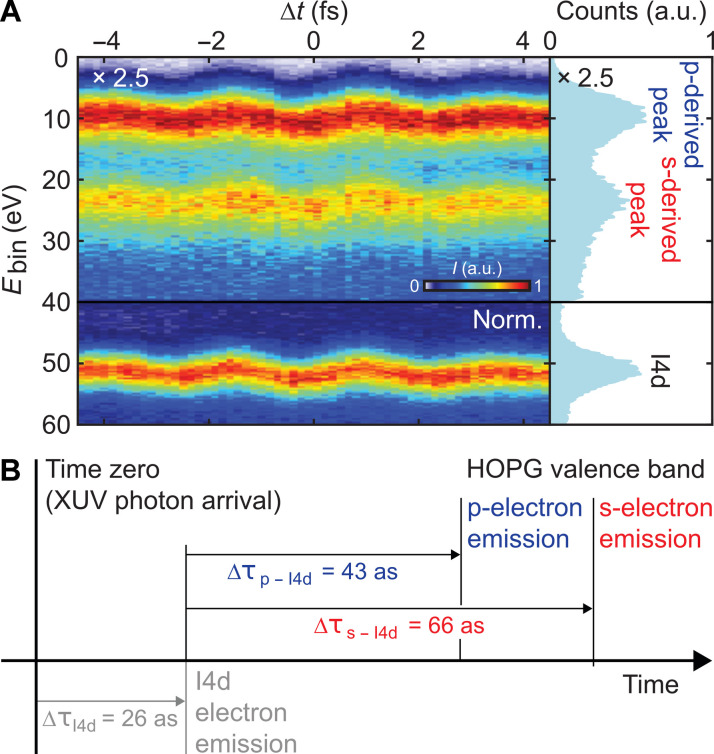
Attosecond chronoscopy on HOPG with an iodoethane monolayer on the surface. (**A**) Attosecond streaking spectrogram recorded at an XUV energy of 105 eV. The two characteristic p- and s-derived traces of the HOPG valence band and the I4d core level trace of the iodoethane monolayer are clearly distinguishable. (**B**) Full time sequence of emission including the photoemission time of I4d as chronoscopic reference (*21*).

Going beyond relative streaking delays between different valence-band components, additional measurements were carried out with the aim of estimating the absolute photoemission time delay of the HOPG valence band relative to the arrival of the XUV pulse. Since Ossiander *et al.* (*21*) identified the absolute photoemission time of the I4d core level of iodoethane, this delay could now be used as a time marker for determining the absolute photoemission time. For this purpose, a monolayer of iodoethane was adsorbed on the HOPG surface and characterized (see Materials and Methods). Measurements at *E*_XUV_ = 105 eV allow the determination of the relative photoemission times between the two characteristic regions of the HOPG valence band (p-derived and s-derived electrons) and the I4d core level to be ∆τ_p−I4d_ = 42.5 ± 11.6 as and ∆τ_s−I4d_ = 66.0 ± 11.4 as (see Fig. 4B). The corresponding absolute photoionization time delay (relative to the arrival time of the peak of the XUV pulse) of ∆τ_p−peak_ = 68.5 ± 12.0 as and ∆τ_p−peak_ = 92.0 ± 11.8 as is determined by adding the absolute photoemission time of I4d. Note that the relative time delays between p-electrons and s-electrons of the HOPG valence band remains almost unchanged by the deposition of the adlayer of iodoethane on the surface providing an independent confirmation of the interpretation of our data. The main uncertainty of our simulation of absolute delays results from the uncertainty of the IMFP which enters the transport time between emission in HOPG and the iodoethane adlayer. Potential orientation-dependent intramolecular time delay contributions from iodoethane (*63*) are expected to be small for large electron energies. The resulting theoretical values $∆\tau_{p-\text{peak}}^{\text{th}}=65\pm 20\;\text{as}$ and $∆\tau_{s-\text{peak}}^{\text{th}}=85\pm 20\;\text{as}$ using the IMFP from (*62*) are in remarkably good quantitative agreement with the experimental data.

## DISCUSSION

In summary, we have experimentally and theoretically demonstrated the link between band structure and EWS delays for dielectric crystalline materials with pronounced modulations of their DOS in the high-energy continuum using attosecond chronoscopy. As the probing NIR field can partially penetrate HOPG along the $\hat{c}$ axis, the EWS time delay τ_EWS_ characterizing the continuum states in the band structure becomes directly accessible. We have determined the difference in EWS delay between electrons emitted from the p- and s-component of the HOPG valence band. Adsorbing a chronoscope atom calibrated by He, the absolute streaking time delay relative to the arrival of the XUV pulse peak could be determined. Both relative and absolute time shifts are in good qualitative accord with theoretical simulations. The strong modulation of the relative time delay, in particular near an XUV energy of *E*_XUV_ = 105 eV, reveals the presence of a pronounced wide bandgap near the final state in the high-energy region of the band structure. The photoelectrons experience a substantial phase shift due to scattering at the periodic potential of the crystal leaving a clear mark on the EWS time delay and the “dwell” time (*52*). This effect manifests itself as a retardation of the photoelectrons when their final energy lies near the bandgap of the HOPG band structure along and close to the surface normal. As demonstrated in this work, attosecond chronoscopy can provide information on material properties such as the band structure and on spectral properties that are difficult to access by conventional spectroscopic techniques. Thus, it has direct implications in areas ranging from condensed matter theory to surface (photo)chemistry and the fabrication of future devices (e.g., lithography with high energy photons).

## MATERIALS AND METHODS

### Attosecond streaking spectroscopy setup

For the present photoemission measurements a carrier-envelope phase stable titanium:sapphire laser system was used (*64*–*66*). Two multipass chirped pulse amplifier stages delivered 3.0 mJ at a repetition rate of 4 kHz, centered around λ_0_ = 789 nm, and a FWHM pulse duration of 23 fs (*67*, *68*). Subsequently, a differentially pumped hollow-core fiber assembly for spectral broadening and mode cleaning was installed (*69*), consisting of two waveguiding glass fiber pieces with a total length of 3 m and a core diameter of 500 μm. A gas inlet between the fiber pieces enabled helium to be injected into the fibers and be pumped out through the entrance and exit. Behind it, a tunable pair of glass wedges and a chirped mirror compressor consisting of two pairs of negatively chirped mirrors (*70*, *71*) are installed to compress the spectrally expanded pulses close to their Fourier limit. This resulted in a high-intensity few-cycle NIR laser pulse with 1.5 mJ at 4 kHz and a pulse duration of ~7 fs for the generation of high harmonics (*72*–*74*). The exit of the fiber assembly ended in a differentially pumped ultrahigh vacuum beamline, which allows to reduce the background pressure from ~8 ∙ 10^−3^ mbar after the fiber assembly down to a background pressure of <2 ∙ 10^−10^ mbar in the experimental chamber. For the high harmonic generation (HHG), the collimated few-cycle NIR beam was focused by a concave mirror with a focal length of 75 cm onto a neon-filled ceramic tube with an inner diameter of 1.6 mm. The beam could pass the tube vertically through a 300-μm through-hole. By supplying the tube with neon gas at a pressure between 75 and 110 mbar, high harmonics with a tunable cutoff energy of 80 to 130 eV could be achieved. A spectrally flat high harmonic cutoff was verified with an XUV spectrometer. The desired central energy of the isolated XUV-attosecond pulse could lastly be adjusted by spectral filtering of the cutoff region with a bandpass multilayer mirror (*75*), which was produced within the Munich Centre for Advanced Photonics. To create a delay between the NIR pulse and the XUV pulse, it is necessary to separate them spatially. This was achieved via a circular 150-μm-thick zirconium foil, positioned at a distance of 2 m behind the HHG ceramic gas target which spatially filters the NIR and XUV beam using their different divergence. The actual delay between the XUV excitation pulse and the NIR streaking field was then set by a double mirror assembly located 1.7 m behind the zirconium foil in the experimental chamber. It includes a 25.4-mm NIR reflecting outer mirror with a 5-mm through-hole in the center, in which a multilayer mirror is located, spectrally filtering and reflecting the XUV pulses. Both beams are focused by the double mirror (125 mm) onto the sample and temporally shifted with respect to each other by a closed-loop piezo nanopositioning system (*76*).

### Sample preparation

To ensure a clean sample surface for the presented attosecond chronoscopy experiments, a HOPG crystal was cleaved in situ under ultrahigh vacuum conditions (<2 ∙ 10^−10^ mbar), and five annealing cycles (5 min heating at 1000 K followed by cooling down at a rate <1 K/s) were done. XPS studies were used to confirm the effectiveness of sample preparation and the absence of contaminants. LEED measurements were performed to ensure the surface and crystal structure quality (see also fig. S1).

To prepare a monolayer of iodoethane on the HOPG surface, an amount of gas corresponding to about 1.5 to 2 layers was adsorbed at 80 K sample temperature. The sample was then heated with 0.5 K/s to 150 K, monitoring the thermal desorption of IC_2_H_5_ with a mass spectrometer. Mono- and multilayer desorption maxima were found to be separated by a deep minimum at 150 K for this heating rate. After complete removal of the second layer by this procedure, the sample was cooled to 80 K for further measurements.

### Adjustment of the DFT energy scale

It is well known that for energies well above the Fermi level, DFT obtains correct pattern of the electronic properties but with considerable scale errors of the energy. To fine-adjust the DFT energy scale, we compared a calculated LEED curent-voltage (IV) curve for the (0, 0) reflex with an experimentally obtained IV curve from the HOPG sample. The experiment was performed with a standard three-grid optics (Omicron), and the IV curve has been extracted from screen images recorded with a video camera. The electron’s angle of incidence was 5° off-normal to enable recording of the (0, 0) reflex on the screen adjacent to the central electron gun. For the experimental value of the electron energy, we added the workfunction of the LaB_6_ cathode of the electron gun [2.5 eV; see e.g., (*77*)] to the acceleration voltage applied between a cathode and a sample. The correction factor deduced by this comparison amounted to a value of 17/16.

## Supplementary Materials

### This PDF file includes:

Theoretical background Figs. S1 and S2 References
