# Supplementary material for: Attosecond chronoscopy of the photoemission near a bandgap of a single-element layered dielectric
Source: Sci Adv. 2024 Jun 26;10(26):eado0073. doi: 10.1126/sciadv.ado0073 (PMC11204203; doi:10.1126/sciadv.ado0073)
Supplement: Supplementary file 1 — Theoretical background Figs. S1 and S2 References [file sciadv.ado0073_sm.pdf]

Science Advances
AAAS

Supplementary Materials for

Attosecond chronoscopy of the photoemission near a bandgap of a single-element layered dielectric

Dionysios Potamianos *et al.*

Corresponding author: Reinhard Kienberger, reinhard.kienberger@tum.de;
Dionysios Potamianos, d.potamianos@lmu.de

Sci. Adv. **10**, eado0073 (2024)
DOI: 10.1126/sciadv.ado0073

This PDF file includes:

Theoretical background
Figs. S1 and S2
References

Theoretical background

Time delay and density of states

In the following, we exploit the relation between the modulation of the density of states $\Delta\rho(E) = \rho(E) - \rho_{\text{free}}(E)$ relative to that of a free particle of the same energy $\rho_{\text{free}}(E)$ and the scattering matrix $S(E)$ to determine the energy dependent Eisenbud-Wigner-Smith (EWS) time delay $\tau_{\text{EWS}}(E)$. It describes the time delay between the (peak) arrival time of the ionizing XUV pulse and the formation of the outgoing wavepacket in the crystal unit cell. From $\tau_{\text{EWS}}(E)$ in which the electronic properties of the target material are encoded, the relative streaking delay (Fig. 3C) and the absolute streaking delay (Fig. 4) can be determined. $\Delta\rho(E)$ is related to the S matrix through

$$\Delta\rho(E) = \frac{-i}{2\pi}\frac{d}{dE}\{\log[\det S(E)]\} \qquad \text{Eq. S1}$$

or, equivalently, to that of the time-delay matrix $Q(E)$,

$$Q(E) = -iS^{+}(E)\frac{\partial}{\partial E}S(E) \qquad \text{Eq. S2}$$

as

$$\Delta\rho(E) = \frac{1}{2\pi}TrQ(E) = \frac{1}{\pi}\sum_j \frac{\partial}{\partial E}\delta^j(E) \qquad \text{Eq. S3}$$

In Eq. S3 $\delta^j(E)$ are the scattering eigenphases and $\tau^j_{EWS} = 2\frac{\partial}{\partial E}\delta^j(E)$ are the eigenvalues of $Q(E)$. These relations (Eqs. S1-S3) are also the underpinning of the Friedel sum rule (*53–57*). The relative half-scattering EWS time delay $\Delta\tau_{\text{EWS},h}$ between p- and s-band derived excitation discussed in the main text is therefore estimated to be

$$\Delta\tau_{\text{EWS},h} = \tau_{\text{EWS},h}(\text{p}) - \tau_{\text{EWS},h}(\text{s}) = \pi\left[\Delta\rho\left(\hbar\omega_{\text{XUV}} - \langle E_{\text{p}}\rangle\right) - \Delta\rho\left(\hbar\omega_{\text{XUV}} - \langle E_{\text{s}}\rangle\right)\right] \qquad \text{Eq. S4}$$

Note that the reference density of states, $\rho_{\text{free}}(E)$, largely drops out from the difference (Eq. S4).

We derive $\rho(E)$ from a VASP calculation of the bulk HOPG unit cell including the 150 lowest-energy bands. We use $60\times 60\times 18$ k-points for a fine-grained resolution of the Brillouin zone and carefully tune convergence parameters to ensure that also the higher-energy unoccupied bands are sufficiently converged. We then average the density of states at high energies over a bandwidth of 2 eV. For the occupied low-energy bands, we project the Kohn-Sham-orbitals onto spherical harmonics to assign s- and p- character to the bands, then calculate first moments of the projected occupied s and p density of states to obtain $\langle E(\text{p}_z)\rangle$ and $\langle E(\text{s})\rangle$.

Streaking time and transport for photoemission from dielectrics

The EWS time for half scattering Eq. S4 describes the spread of the initial photoionization wavepacket over the lattice unit cell (2 atomic layers wide in $\hat{c}$-direction) in real space. For an energy dependent inelastic mean free path (IMFP) of electrons in the conduction band, $\lambda(E)$, excited by a photon with energy $E_{XUV} \approx 100$ eV along the $\hat{c}$-direction, the estimated IMFP ranges from about 8.5 a.u. (isotropic calculation (*61*)) to $\lambda \sim 16.75$ a.u. for the direction normal to the atomic planes (*62*). Correspondingly, about $\overline{N}_{\text{vb}} = \lambda_{\text{vb}}/(2\text{d}) \approx 0.75 - 1.3$ unit cells or, equivalently, $1.5 - 2.6$ atomic layers effectively contribute on average. The elastic transport through these layers results in an additional delay which is given by the full scattering delay τ_{EWS}.

We note that when applying Eq. S4 to the difference between p- and s-components of valence band emission, $\Delta\tau_{EWS}$, uncertainties in the layer spacing and mean free path largely cancel out since the relative difference in emission energy, $\Delta E/E \sim 10\%$, of the two valence band contributions is quite small. These uncertainties have, however, a larger impact on absolute delays.

In the experiment, the streaking time delay τ_s rather than the EWS time delay τ_{EWS} or transport time τ_t is directly accessible. For a highly anisotropic dielectric such as HOPG, the streaking IR field can penetrate the layers contributing to Eq. S4, however reduced by dielectric screening $\varepsilon_{\perp}^{-1}(\omega_{IR})$ where $\varepsilon_{\perp}(\omega_{IR})$ is the frequency dependent optical dielectric function along the direction perpendicular to the layers. For HOPG $\varepsilon_{\perp}(\omega_{IR}) \approx 2$ (*43, 44, 78*). The relation between the band structure induced EWS time delay and the streaking-time delay is given for a dielectric by

$$\Delta t_s = \omega_{IR}^{-1}\tan^{-1}\left[\frac{\sin\left[\omega_{IR}\left(\tau_{EWS}+\tau_{CLC}\right)\right]+\left(\varepsilon_{\perp}-1\right)\sin\left(\omega_{IR}\tau_t\right)}{\cos\left[\omega_{IR}\left(\tau_{EWS}+\tau_{CLC}\right)\right]+\left(\varepsilon_{\perp}-1\right)\cos\left(\omega_{IR}\tau_t\right)}\right] \qquad \text{Eq. S5}$$

where we have included for completeness the Coulomb-laser coupling induced time shift τ_{CLC} accounting for the interplay between the Coulomb field of the ionic core and the streaking IR field and the transport time τ_t (*5, 22, 36, 37*) of a quasi-free electron, $\tau_t = \lambda(E)/v(E)$, from the point of photoexcitation to the surface. While in the present case of emission of fast electrons ($E_{XUV} \approx 100$ eV) the CLC-contribution ($\tau_{CLC} < 5$ as) as well as image charge effects are negligible, the transport time enters the absolute time delays determined by reference to chronoscopic molecules deposited at the surface (see main text).

Using the fact that in the present case EWS delays as well as transport times are short on the time scale of the cycle period of the IR field, $|\omega_{IR}\tau_{EWS}| \ll 1$ and $|\omega_{IR}\tau_t| \ll 1$, Eq. S5 can be linearized to yield

$$t_s \approx \frac{\tau_{EWS} + \left(\varepsilon_{\perp}-1\right)\tau_t}{\varepsilon_{\perp}} \qquad \text{Eq. S6}$$

Relative streaking delays between different bulk processes of a dielectric are therefore given in terms of the differences in EWS delays only,

$$\Delta t_s \approx \Delta\tau_{EWS}/\varepsilon_{\perp} \qquad \text{Eq. S7}$$

while in the limit of a conductor ($\varepsilon_{\perp} \rightarrow \infty$) streaking times reduce to the transport (or arrival) time at the surface (*5, 22, 36, 37*)

$$t_s \approx \tau_t \qquad \text{Eq. S8}$$

Eq. S6 provides the underpinning to the simulations of the relative time delays between different bulk-emission processes (from the s- and p-derived bands) as well as of the absolute time delays clocked by referring to chronoscope molecules adsorbed at the surface (see main text). It should be noted that the comparison with the experimental data for both relative and absolute time delays involves no freely adjustable parameter.

EWS-delay from scattering states

Complementing our investigations of time delays based on the connection between τ_{EWS} and the modulation of the density of states (Eqs. S1-S3) for the realistic 3d band structure of HOPG, we have also explored the connection between τ_{EWS} and the spectral variation of the scattering phase of inverse LEED states $\tau_{\mathrm{EWS}} = \frac{d}{dE}\delta(E)$ for a one-dimensional model of HOPG. For this system we employ a one-dimensional ground-state DFT potential for the lattice generated by averaging over planes parallel to the surface. Such a simplified 1d-model faces the difficulty that the AB stacking cannot be properly represented by the planar-averaged potential $V(z)$. Consequently, the length of the unit cell in the 1d-model representing the z-direction is 3.35 Å rather than 6.7 Å as in the 3d DFT simulation. Correspondingly, the Brillouin zone is twice as large and the band gaps appear now at $E_{n'} n'^{2} \cdot 3.35$ eV and, thus, at different positions. The gaps energetically closest to the experiment ($n = 11$ in 3d) are near 66 eV ($E_{n'=5}$) and 103 eV ($E_{n'=6}$). We emphasize that the numerical results of this model are not expected to closely resemble the results of the full 3d calculation. However, the qualitative behavior of τ_{EWS} near such a band gap is unaffected by this mismatch between the 3d- and 1d-models for the band gap position and can therefore provide qualitative insights into the impact of band gaps on the delays and, more generally, into the relation between spectral modulation of DOS and of scattering phases.

The ground state of a 13 layer slab is excited by the XUV pulse to a high-lying state in the continuum and the resulting wavepacket is propagated for 200 a.u. (~ 4.8 fs) subsequent to the conclusion of the pulse. Inelastic scattering and the finite escape depth of photoelectrons are accounted for by using an (optical) imaginary potential (*37*). τ_{EWS} can be determined by the difference in arrival time of the wavepacket the potential landscape at a virtual detector far from the surface (solid line) relative to that of the reference wavepacket of a free electron (dashed line). In Fig. S2 the difference between the p- and s-derived electrons (in the 1d-model only distinguished by the different kinetic energies) is shown. The simulation clearly displays the modulation of the EWS time delay difference $\tau_{\mathrm{p}} - \tau_{\mathrm{s}}$ when the XUV energy is scanned over the spectral region of the band gap in qualitative accord with the difference in dwell time discussed above and with the experiment.

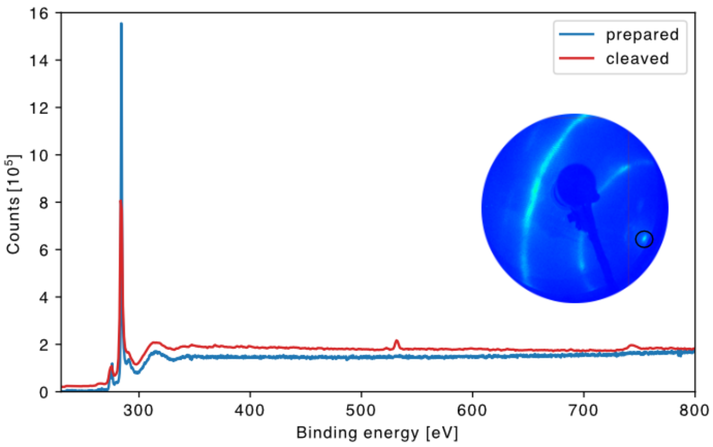

Fig. S1: XPS and LEED characterizations of HOPG. Red line: XPS after cleaving in UHV. Blue line: XPS after cleaving and five cycles of annealing. The absence of the O1s peak (~530 eV) is indicative of the low contamination of the surface. Inset: LEED image where the HOPG crystal is tilted to reveal the 0th order diffraction spot (highlighted by the black circle), while the diffracted electrons form a ring pattern. The LEED image indicates the highly oriented, polycrystalline nature of the sample with crystalline regions of various rotations around the surface normal.

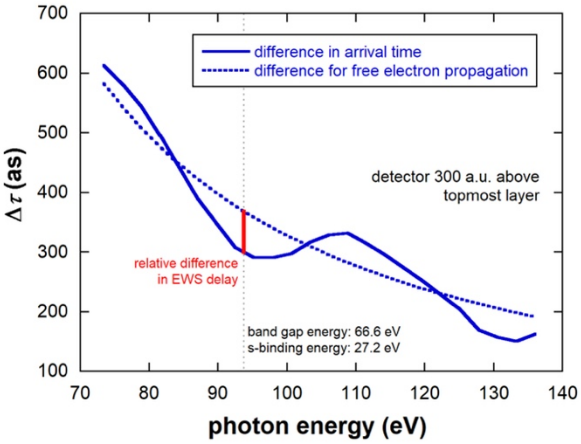

Fig. S2: Time difference of arrival of p-electrons and s-electrons at a virtual detector positioned 300 a.u. above the surface. Dotted line: expected arrival difference for free electrons, solid line: calculated time difference after photoexcitation in 13-layer HOPG slab. The vertical dotted line indicates the photon energy at which s-electrons cross the 1d band gap ($n' = 5$) at 66.6 eV.

REFERENCES AND NOTES

1. A. Damascelli, Probing the electronic structure of complex systems by ARPES. *Phys. Scr.* **T109**, 61–74 (2004).

2. C. Jozwiak, C. H. Park, K. Gotlieb, C. Hwang, D. H. Lee, S. G. Louie, J. D. Denlinger, C. R. Rotundu, R. J. Birgeneau, Z. Hussain, A. Lanzara, Photoelectron spin-flipping and texture manipulation in a topological insulator. *Nat. Phys.* **9**, 293–298 (2013).

3. J. H. Ryoo, C. H. Park, Spin-conserving and reversing photoemission from the surface states of Bi_2Se_3 and Au (111). *Phys. Rev. B* **93**, 085419 (2016).

4. J. Krempaský, M. Fanciulli, L. Nicolaï, J. Minár, H. Volfová, O. Caha, V. V. Volobuev, J. Sánchez-Barriga, M. Gmitra, K. Yaji, K. Kuroda, S. Shin, F. Komori, G. Springholz, J. H. Dil, Fully spin-polarized bulk states in ferroelectric GeTe. *Phys. Rev. Res.* **2**, 013107 (2020).

5. R. Pazourek, S. Nagele, J. Burgdörfer, Attosecond chronoscopy of photoemission. *Rev. Mod. Phys.* **87**, 765–802 (2015).

6. H. G. Muller, Reconstruction of attosecond harmonic beating by interference of two-photon transitions. *Appl. Phys. B* **74**, s17–s21 (2002).

7. R. Kienberger, E. Goulielmakis, M. Uiberacker, A. Baltuska, V. Yakovlev, F. Bammer, A. Scrinzi, T. Westerwalbesioh, U. Kleineberg, U. Heinzmann, M. Drescher, F. Krausz, Atomic transient recorder. *Nature* **427**, 817–821 (2004).

8. C. Cirelli, M. Sabbar, S. Heuser, R. Boge, M. Lucchini, L. Gallmann, U. Keller, Energy-dependent photoemission time delays of noble gas atoms using coincidence attosecond streaking. *IEEE J. Sel. Top. Quantum Electron.* **21**, 8700307 (2015).

9. C. H. Zhang, U. Thumm, Streaking and Wigner time delays in photoemission from atoms and surfaces. *Phys. Rev. A* **84**, 033401 (2011).

10. S. Nagele, R. Pazourek, J. Feist, K. Doblhoff-Dier, C. Lemell, K. Tókési, J. Burgdörfer, Time-resolved photoemission by attosecond streaking: Extraction of time information. *J. Phys. B: At. Mol. Opt. Phys.* **44**, 081001 (2011).

11. P. Eckle, M. Smolarski, P. Schlup, J. Biegert, A. Staudte, M. Schöffler, H. G. Muller, R. Dörner, U. Keller, Attosecond angular streaking. *Nat. Phys.* **4**, 565–570 (2008).

12. S. Li, T. Driver, P. Rosenberger, E. G. Champenois, J. Duris, A. Al-Haddad, V. Averbukh, J. C. T. Barnard, N. Berrah, C. Bostedt, P. H. Bucksbaum, R. N. Coffee, L. F. DiMauro, L. Fang, D. Garratt, A. Gatton, Z. Guo, G. Hartmann, D. Haxton, W. Helml, Z. Huang, A. C. LaForge, A. Kamalov, J. Knurr, M.-F. Lin, A. A. Lutman, J. P. MacArthur, J. P. Marangos, M. Nantel, A. Natan, R. Obaid, J. T. O'Neal, N. H. Shivaram, A. Schori, P. Walter, A. L. Wang, T. J. A. Wolf, Z. Zhang, M. F. Kling, A. Marinelli, J. P. Cryan, Attosecond coherent electron motion in Auger-Meitner decay. *Science* **375**, 285–290 (2022).

13. M. Fanciulli, H. Volfová, S. Muff, J. Braun, H. Ebert, J. Minár, U. Heinzmann, J. H. Dil, Spin polarization and attosecond time delay in photoemission from spin degenerate states of solids. *Phys. Rev. Lett.* **118**, 067402 (2017).

14. T. Barillot, C. Cauchy, P. A. Hervieux, M. Gisselbrecht, S. E. Canton, P. Johnsson, J. Laksman, E. P. Mansson, J. M. Dahlström, M. Magrakvelidze, G. Dixit, M. E. Madjet, H. S. Chakraborty, E. Suraud, P. M. Dinh, P. Wopperer, K. Hansen, V. Loriot, C. Bordas, S. Sorensen, F. Lépine, Angular asymmetry and attosecond time delay from the giant plasmon resonance in C_{60} photoionization. *Phys. Rev. A* **91**, 033413 (2015).

15. F. Roth, T. Arion, H. Kaser, A. Gottwald, W. Eberhardt, Angle resolved Photoemission from Ag and Au single crystals: Final state lifetimes in the attosecond range. *J. Electron. Spectros. Relat. Phenomena* **224**, 84–92 (2018).

16. Z. Tao, C. Chen, T. Szilvási, M. Keller, M. Mavrikakis, H. Kapteyn, M. Murnane, Direct time-domain observation of attosecond final-state lifetimes in photoemission from solids. *Science* **353**, 62–67 (2016).

17. M. Ossiander, F. Siegrist, V. Shirvanyan, R. Pazourek, A. Sommer, T. Latka, A. Guggenmos, S. Nagele, J. Feist, J. Burgdörfer, R. Kienberger, M. Schultze, Attosecond correlation dynamics. *Nat. Phys.* **13**, 280–285 (2017).

18. M. Isinger, R. J. Squibb, D. Busto, S. Zhong, A. Harth, D. Kroon, S. Nandi, C. L. Arnold, M. Miranda, J. M. Dahlström, E. Lindroth, R. Feifel, M. Gisselbrecht, A. L'Huillier, Photoionization in the time and frequency domain. *Science* **358**, 893–896 (2017).

19. J. Feist, O. Zatsarinny, S. Nagele, R. Pazourek, J. Burgdörfer, X. Guan, K. Bartschat, B. I. Schneider, Time delays for attosecond streaking in photoionization of neon. *Phys. Rev. A* **89**, 033417 (2014).

20. R. Locher, L. Castiglioni, M. Lucchini, M. Greif, L. Gallmann, J. Osterwalder, M. Hengsberger, U. Keller, Energy-dependent photoemission delays from noble metal surfaces by attosecond interferometry. *Optica* **2**, 405 (2015).

21. M. Ossiander, J. Riemensberger, S. Neppl, M. Mittermair, M. Schäffer, A. Duensing, M. S. Wagner, R. Heider, M. Wurzer, M. Gerl, M. Schnitzenbaumer, J. V. Barth, F. Libisch, C. Lemell, J. Burgdörfer, P. Feulner, R. Kienberger, Absolute timing of the photoelectric effect. *Nature* **561**, 374–377 (2018).

22. A. L. Cavalieri, N. Müller, T. Uphues, V. S. Yakovlev, A. Baltuška, B. Horvath, B. Schmidt, L. Blümel, R. Holzwarth, S. Hendel, M. Drescher, U. Kleineberg, P. M. Echenique, R. Kienberger, F. Krausz, U. Heinzmann, Attosecond spectroscopy in condensed matter. *Nature* **449**, 1029–1032 (2007).

23. S. Neppl, R. Ernstorfer, E. M. Bothschafter, A. L. Cavalieri, D. Menzel, J. V. Barth, F. Krausz, R. Kienberger, P. Feulner, Attosecond time-resolved photoemission from core and valence states of magnesium. *Phys. Rev. Lett.* **109**, 087401 (2012).

24. Q. Liao, U. Thumm, Attosecond time-resolved photoelectron dispersion and photoemission time delays. *Phys. Rev. Lett.* **112**, 023602 (2014).

25. C. Lemell, S. Neppl, G. Wachter, K. Tokési, R. Ernstorfer, P. Feulner, R. Kienberger, J. Burgdörfer, Real-time observation of collective excitations in photoemission. *Phys. Rev. B* **91**, 241101 (2015).

26. A. G. Borisov, D. Sánchez-Portal, A. K. Kazansky, P. M. Echenique, Resonant and nonresonant processes in attosecond streaking from metals. *Phys. Rev. B* **87**, 121110 (2013).

27. J. Riemensberger, S. Neppl, D. Potamianos, M. Schäffer, M. Schnitzenbaumer, M. Ossiander, C. Schröder, A. Guggenmos, U. Kleineberg, D. Menzel, F. Allegretti, J. V. Barth, R. Kienberger, P. Feulner, A. G. Borisov, P. M. Echenique, A. K. Kazansky, Attosecond dynamics of sp-band photoexcitation. *Phys. Rev. Lett.* **123**, 176801 (2019).

28. L. Kasmi, M. Lucchini, L. Castiglioni, P. Kliuiev, J. Osterwalder, M. Hengsberger, L. Gallmann, P. Krüger, U. Keller, Effective mass effect in attosecond electron transport. *Optica* **4**, 1492 (2017).

29. C. Chen, Z. Tao, A. Carr, P. Matyba, T. Szilvási, S. Emmerich, M. Piecuch, M. Keller, D. Zusin, S. Eich, M. Rollinger, W. You, S. Mathias, U. Thumm, M. Mavrikakis, M. Aeschlimann, P. M. Oppeneer, H. Kapteyn, M. Murnane, Distinguishing attosecond electron-electron scattering and screening in transition metals. *Proc. Natl. Acad. Sci. U.S.A.* **114**, E5300–E5307 (2017).

30. M. Volkov, S. A. Sato, F. Schlaepfer, L. Kasmi, N. Hartmann, M. Lucchini, L. Gallmann, A. Rubio, U. Keller, Attosecond screening dynamics mediated by electron localization in transition metals. *Nat. Phys.* **15**, 1145–1149 (2019).

31. F. Siek, S. Neb, P. Bartz, M. Hensen, C. Strüber, S. Fiechter, M. Torrent-Sucarrat, V. M. Silkin, E. E. Krasovskii, N. M. Kabachnik, S. Fritzsche, R. D. Muiño, P. M. Echenique, A. K. Kazansky, N. Müller, W. Pfeiffer, U. Heinzmann, Angular momentum–induced delays in solid-state photoemission enhanced by intra-atomic interactions. *Science* **357**, 1274–1277 (2017).

32. E. E. Krasovskii, Character of the outgoing wave in soft x-ray photoemission. *Phys. Rev. B* **102**, 245139 (2020).

33. S. Neppl, R. Ernstorfer, A. L. Cavalieri, C. Lemell, G. Wachter, E. Magerl, E. M. Bothschafter, M. Jobst, M. Hofstetter, U. Kleineberg, J. V. Barth, D. Menzel, J. Burgdörfer, P. Feulner, F. Krausz, R. Kienberger, Direct observation of electron propagation and dielectric screening on the atomic length scale. *Nature* **517**, 342–346 (2015).

34. M. Y. Amusia, A. S. Baltenkov, Time delay of slow electrons-endohedral elastic scattering. *Fuller. Nanotub. Carbon Nanostruct.* **28**, 10–13 (2020).

35. L. Seiffert, Q. Liu, S. Zherebtsov, A. Trabattoni, P. Rupp, M. C. Castrovilli, M. Galli, F. Süßmann, K. Wintersperger, J. Stierle, G. Sansone, L. Poletto, F. Frassetto, I. Halfpap, V. Mondes, C. Graf, E. Rühl, F. Krausz, M. Nisoli, T. Fennel, F. Calegari, M. F. Kling, Attosecond chronoscopy of electron scattering in dielectric nanoparticles. *Nat. Phys.* **13**, 766–770 (2017).

36. C. Lemell, B. Solleder, K. Tokési, J. Burgdörfer, Simulation of attosecond streaking of electrons emitted from a tungsten surface. *Phys. Rev. A* **79**, 062901 (2009).

37. E. E. Krasovskii, C. Friedrich, W. Schattke, P. M. Echenique, Rapid propagation of a Bloch wave packet excited by a femtosecond ultraviolet pulse. *Phys. Rev. B* **94**, 195434 (2016).

38. R. O. Kuzian, E. E. Krasovskii, One-step theory of photoelectron escape time: Attosecond spectroscopy of Mg(0001). *Phys. Rev. B* **102**, 115116 (2020).

39. J. Pappis, S. L. Blum, Properties of pyrolytic graphite. *J. Am. Ceram. Soc.* **44**, 592–597 (1961).

40. L. C. F. Blackman, A. R. J. P. Ubbelohde, Stress recrystallization of graphite. *Proc. R. Soc. Lond. A Math. Phys. Sci.* **266**, 20–32 (1962).

41. S. Kawai, H. Kawakatsu, Surface-relaxation-induced giant corrugation on graphite (0001). *Phys. Rev. B* **79**, 115440 (2009).

42. F. Matsui, H. Nishikawa, H. Daimon, M. Muntwiler, M. Takizawa, H. Namba, T. Greber, The 4π k_z periodicity in photoemission from graphite. *Phys. Rev. B* **97**, 045430 (2018).

43. A. B. Djurišić, E. H. Li, Optical properties of graphite. *J. Appl. Phys.* **85**, 7404–7410 (1999).

44. B. Song, H. Gu, S. Zhu, H. Jiang, X. Chen, C. Zhang, S. Liu, Broadband optical properties of graphene and HOPG investigated by spectroscopic Mueller matrix ellipsometry. *Appl. Surf. Sci.* **439**, 1079–1087 (2018).

45. C. Lechner, B. Pannier, P. Baranek, N. C. Forero-Martinez, H. Vach, First-principles study of the structural, electronic, dynamic, and mechanical properties of HOPG and diamond: Influence of exchange-correlation functionals and dispersion interactions. *J. Phys. Chem. C* **120**, 5083–5100 (2016).

46. K. Endo, S. Koizumi, T. Otsuka, M. Suhara, T. Morohasi, E. Z. Kurmaev, D. P. Chong, Analysis of XPS and XES of diamond and graphite by DFT calculations using model molecules. *J. Comput. Chem.* **22**, 102–108 (2001).

47. M. Schultze, M. Fieß, N. Karpowicz, J. Gagnon, M. Korbman, M. Hofstetter, S. Neppl, A. L. Cavalieri, Y. Komninos, T. Mercouris, C. A. Nicolaides, R. Pazourek, S. Nagele, J. Feist, J. Burgdörfer, A. M. Azzeer, R. Ernstorfer, R. Kienberger, U. Kleineberg, E. Goulielmakis, F. Krausz, V. S. Yakovlev, Delay in photoemission. *Science* **328**, 1658–1662 (2010).

48. J. Gagnon, E. Goulielmakis, V. S. Yakovlev, The accurate FROG characterization of attosecond pulses from streaking measurements. *Appl. Phys. B* **92**, 25–32 (2008).

49. M. Lucchini, M. H. Brügmann, A. Ludwig, L. Gallmann, U. Keller, T. Feurer, Ptychographic reconstruction of attosecond pulses. *Opt. Express* **23**, 29502–29513 (2015).

50. M. Lucchini, M. Nisoli, Refined ptychographic reconstruction of attosecond pulses. *Appl. Sci.* **8**, 2563 (2018).

51. H. Wei, T. Morishita, C. D. Lin, Critical evaluation of attosecond time delays retrieved from photoelectron streaking measurements. *Phys. Rev. A* **93**, 053412 (2016).

52. G. Iannaccone, General relation between density of states and dwell times in mesoscopic systems. *Phys Rev B* **51**, 4727–4729 (1995).

53. J. S. Faulkner, Scattering theory and cluster calculations. *J. Phys. C: Solid State Phys.* **10**, 4661–4670 (1977).

54. J. Friedel, XIV. XIV. The distribution of electrons round impurities in monovalent metals. *Science* **43**, 153–189 (1952).

55. J. Friedel, Electronic structure of primary solid solutions in metals. *Adv. Phys.* **3**, 446–507 (1954).

56. J. Friedel, Structure électronique des impuretés dans les métaux. *Ann. Phys.* **12**, 158–202 (1954).

57. J. Friedel, Metallic alloys. *Il Nuovo Cimento* **7**, 287–311 (1958).

58. C. Kittel, *Quantum Theory of Solids* (Wiley, ed. 2, 1991).

59. B. V. Chirikov, F. M. Izrailev, D. L. Shepelyansky, Quantum chaos: Localization vs. ergodicity. *Physica D* **33**, 77–88 (1988).

60. Č. Lozej, Spectral form factor and dynamical localization. *Entropy* **25**, 451 (2023).

61. S. Tanuma, C. J. Powell, D. R. Penn, Calculations of electron inelastic mean free paths. IX. Data for 41 elemental solids over the 50 eV to 30 keV range. *Surf. Interface Anal.* **43**, 689–713 (2011).

62. M. Azzolini, T. Morresi, K. Abrams, R. Masters, N. Stehling, C. Rodenburg, N. M. Pugno, S. Taioli, M. Dapor, Anisotropic approach for simulating electron transport in layered materials: Computational and experimental study of highly oriented pyrolitic graphite. *J. Phys. Chem. C* **122**, 10159–10166 (2018).

63. S. Biswas, B. Förg, L. Ortmann, J. Schötz, W. Schweinberger, T. Zimmermann, L. Pi, D. Baykusheva, H. A. Masood, I. Liontos, A. M. Kamal, N. G. Kling, A. F. Alharbi, M. Alharbi, A. M. Azzeer, G. Hartmann, H. J. Wörner, A. S. Landsman, M. F. Kling, Probing molecular environment through photoemission delays. *Nat. Phys.* **16**, 778–783 (2020).

64. H. R. Telle, G. Steinmeyer, A. E. Dunlop, J. Stenger, D. H. Sutter, U. Keller, Carrier-envelope offset phase control: A novel concept for absolute optical frequency measurement and ultrashort pulse generation. *Appl. Phys. B* **69**, 327–332 (1999).

65. C. Spielmann, P. F. Curley, T. Brabec, F. Krausz, Ultrabroadband femtosecond lasers. *IEEE J. Quantum. Electron.* **30**, 1100–1114 (1994).

66. L. Xu, T. W. Hänsch, Ch. Spielmann, A. Poppe, T. Brabec, F. Krausz, Route to phase control of ultrashort light pulses. *Opt. Lett.* **21**, 2008–2010 (1996).

67. D. Strickland, G. Mourou, Compression of amplified chirped optical pulses. *Opt. Commum.* **56**, 219–221 (1985).

68. S. Sartania, Z. Cheng, M. Lenzner, G. Tempea, C. Spielmann, F. Krausz, K. Ferencz, Generation of 01-TW 5-fs optical pulses at a 1-kHz repetition rate. *Opt. Lett.* **22**, 1562–1564 (1997).

69. M. Nurhuda, A. Suda, K. Midorikawa, H. Budiono, Control of self-phase modulation and plasma-induced blueshifting of high-energy, ultrashort laser pulses in an argon-filled hollow fiber using conjugate pressure-gradient method. *J. Opt. Soc. Am. B* **22**, 1757 (2005).

70. V. Pervak, A. V. Tikhonravov, M. K. Trubetskov, S. Naumov, F. Krausz, A. Apolonski, 1.5-octave chirped mirror for pulse compression down to sub-3 fs. *Appl. Phys. B* **87**, 5–12 (2007).

71. R. Szipöcs, C. Spielmann, F. Krausz, K. Ferencz, Chirped multilayer coatings for broadband dispersion control in femtosecond lasers. *Opt. Lett.* **19**, 201 (1994).

72. A. McPherson, G. Gibson, H. Jara, U. Johann, T. S. Luk, I. A. McIntyre, K. Boyer, C. K. Rhodes, Studies of multiphoton production of vacuum-ultraviolet radiation in the rare gases. *J. Opt. Soc. Am. B* **4**, 595 (1987).

73. M. Lewenstein, P. Balcou, M. Y. Ivanov, A. L'Huillier, P. B. Corkum, Theory of high-harmonic generation by low-frequency laser fields. *Phys. Rev. A* **49**, 2117–2132 (1994).

74. P. B. Corkum, Plasma perspective on strong field multiphoton ionization. *Phys. Rev. Lett.* **71**, 1994–1997 (1993).

75. R. Kienberger, M. Hentschel, C. Spielmann, G. A. Reider, N. Milosevic, U. Heinzmann, M. Drescher, F. Krausz, Sub-femtosecond X-ray pulse generation and measurement. *Appl. Phys. B* **74**, s3–s9 (2002).

76. E. Magerl, S. Neppl, A. L. Cavalieri, E. M. Bothschafter, M. Stanislawski, T. Uphues, M. Hofstetter, U. Kleineberg, J. V. Barth, D. Menzel, F. Krausz, R. Ernstorfer, R. Kienberger, P. Feulner, A flexible apparatus for attosecond photoelectron spectroscopy of solids and surfaces. *Rev. Sci. Instrum.* **82**, 063104 (2011).

77. V. Katsap, LaB6 cathode workfunction evaluation. *2013 IEEE 14th International Vacuum Electronics Conference (IVEC)*, (2013); 10.1109/IVEC.2013.6570897.

78. G. E. Jellison, J. D. Hunn, H. N. Lee, Measurement of optical functions of highly oriented pyrolytic graphite in the visible. *Phys. Rev. B* **76**, 085125 (2007).
